# Superior antiviral and antiproliferative activity of IFN-beta vs. IFN-alpha in primary ATL cells occurs downstream of STAT1 signaling

**DOI:** 10.1186/1742-4690-11-S1-O22

**Published:** 2014-01-07

**Authors:** Ricardo Khouri, Gilvaneia Silva-Santos, Lourdes Farre, Achilea Bittencourt, Anne-Mieke Vandamme, Johan Van Weyenbergh

**Affiliations:** 1 Rega Institute for Medical Research, Department of Microbiology and Immunology, K.U.Leuven, Belgium; 2 CPqGM-FIOCRUZ, Salvador-Bahia, Brazil; 3 HUPES-UFBA, Salvador-BA, Brazil; 4 Centro de Malária e outras Doenças Tropicais and Unidade de Microbiologia, Instituto de Higiene e Medicina Tropical, Universidade Nova de Lisboa, Portugal; 5 Institute for Immunological Investigation, iii-INCT, Brazil

## 

Adult T-cell leukemia (ATL) is an aggressive CD4^+^CD25^+^ leukemia with poor prognosis, which usually develops several decades after HTLV-1 infection. In contrast to HIV-infection, the treatment of HTLV-1-associated diseases rely on a limited number of drugs. For ATL, combination therapy with IFN-alpha+AZT has shown clinical benefit in the non-lymphoma subtypes. Type I IFNs (IFN-alpha/beta) are essential cytokines with proved anti-cancer and antiviral action in vitro and in vivo. Nonetheless, their mechanisms of action in HTLV-1 infection remain unclear and a side-by-side comparison of both type I IFNs has not been performed in ATL. We show, in short-term culture of primary mononuclear cells from ATL patients, that both IFNs cause increased apoptosis, exert an anti-proliferative and antiviral effect, and decrease pro-inflammatory cytokine levels. However, IFN-beta treatment was significantly more effective in inhibiting viral p19 protein levels and lymphoproliferation, as compared to IFN-alpha. This pronounced effect of IFN-beta was explained by an induction of a higher number of known IFN-stimulated genes and antiviral genes by microarray analysis (76 vs. 26 genes were selected with p<0.001 and >2-fold difference vs. control). In PBMCs from healthy donors, ATL patients as well as in HTLV-1-infected cell lines, both IFNs have comparable activity in phosphorylating STATs 1 through 5 (PhosFlow), although phospho-STAT1 levels were up to tenfold higher than phospho-STAT2 through 5. This predominant STAT1-mediated antiviral gene signature was confirmed by Ingenuity Pathway analysis. In conclusion, our data suggest the superior antiviral and antiproliferative activity of IFN-beta vs. IFN-alpha occurs downstream of STAT1 signaling.

